# In Situ Structural Characterization of Cardiomyocyte Microenvironment by Multimodal STED Microscopy

**DOI:** 10.3390/photonics11060533

**Published:** 2024-06-03

**Authors:** Zhao Zhang, Bruce Z. Gao, Tong Ye

**Affiliations:** 1 Department of Bioengineering, Clemson University, Clemson, SC 29634, USA;; 2 Department of Regenerative Medicine and Cell Biology, Medical University of South Carolina, Charleston, SC 29425, USA

**Keywords:** STED microscopy, SHG microscopy, quantitative microscopy, immunofluorescence, autofluorescence, myocardium

## Abstract

Within the myocardium, cardiomyocytes reside in a complex and dynamic extracellular matrix (ECM) consisting of a basement membrane (BM) and interstitial matrix. The interactions between cardiomyocytes and the myocardial ECM play a critical role in maintaining cardiac geometry and function throughout cardiac development and in adult hearts. Understanding how the structural changes of the myocardial ECM affect cardiomyocyte function requires knowledge of pericellular structures. These structures are of a size beyond the resolution of conventional optical microscopy. Here, we demonstrated multi-scale and multi-aspect characterization of the cardiomyocyte microenvironment in myocardial tissue sections using multimodal stimulated emission depletion (STED) microscopy. Second harmonic generation and autofluorescence facilitated multiplexed imaging, enabling the interpretation of protein distribution in 3D. STED imaging modality revealed BM structures of cardiomyocytes and myocardial capillaries at the subdiffractional level. Moreover, meaningful measurements retrieved from acquired images, such as sarcomere length and capillary density, enabled quantitative assessment of myocardial structures.

## 1. Introduction

Cardiomyopathy, a disease of the heart muscle, is characterized by structural and functional abnormalities of the myocardium in the absence of coronary artery disease, hypertension, valvular disease, or congenital heart disease. In 2020, there were approximately 6.11 million prevalent cases of cardiomyopathy and myocarditis and 0.37 million related deaths worldwide. Many affected individuals are asymptomatic, and chronically treated patients are at risk of heart failure. Within the myocardium, cardiomyocytes reside in a complex and dynamic extracellular matrix (ECM) consisting of a basement membrane (BM) and interstitial matrix. The interactions between cardiomyocytes and the myocardial ECM play a critical role in maintaining cardiac geometry and function throughout cardiac development and in adult hearts. Evidence from patient and animal models has demonstrated that abnormal alterations of myocardial ECM composition and nanostructure are pathogenic and associated with cardiomyopathy. Understanding how the structural changes of the myocardial ECM affect cardiomyocyte function requires knowledge of BM structures in the pericellular domain.

Electron microscopy (EM) can perform ultrastructural analysis of the basement membrane [1–4]; however, its application in studying cell environments in tissue samples is challenging due to the critical requirement of sample thickness, the lack of molecular specificity, and the difficulty of 3D characterization. Since the thickness is typically below 100 nm in human heart tissues characterized by EM [3,5], imaging beyond the resolution limit (~200 nm) is required to characterize the cardiomyocyte microenvironment. Fluorescence resonance energy transfer microscopy (FRET) has been used for visualizing and quantifying cell-microenvironment interactions, particularly in the investigation of mechanotransduction associated with cell adhesion and cell migration [6–8]. However, FRET is currently limited to the studies of dissociated cell cultures on 2D substrates or in 3D in vitro models due to the difficulties of utilizing FRET biosensors in vivo [6,8,9]. Total internal reflection fluorescence microscopy (TIRF) can be used to image structures in the vicinity of cell membranes by an evanescent field, which confines the excitation within a region of ~100 nm in thickness. TIRF-based single-molecule localization microscopy, photoactivated localization microscopy (PALM), and stochastic optical reconstruction microscopy (STORM) can achieve a typical resolution of ~20 nm, enabling the nanoscale organization within BM structures to be revealed. However, the limited penetration depth of the evanescent field impedes the visualization of structures deep within a sample, essentially a micrometer-thick tissue section. There is a lack of effective tools that allow for in situ characterization and assessment of cardiomyocyte microenvironment at the tissue level.

Stimulated emission depletion (STED) microscopy is one of the most popular super-resolution imaging techniques, inheriting the benefits of confocal microscopy, such as optical sectioning and molecular specificity [10,11]. In addition, STED microscopy outperforms other super-resolution imaging techniques by rapid acquisition, free of computational post-processing for image formation, and the capability to image thick samples with densely packed features [12]. Constrained by the Nyquist sampling criterion, it is inefficient to perform super-resolution imaging of a large field-of-view (FOV). In addition to prolonged acquisition, long-term exposure to intense depletion laser usually causes severe photobleaching, low SNR, and degraded image quality [13]. Pre-determining regions of interest (ROIs) for STED imaging is necessary to preserve effective resolution, reduce frame rate, and minimize photobleaching. Counterstaining is commonly used in fluorescence microscopy to facilitate the interpretation of cellular structures and tissue morphology. Localizing ROIs by counterstaining complicates sample preparation and increases the complexity of a STED system, requiring additional excitation sources and detection channels. Thanks to intrinsic second harmonic generation (SHG) sources in the myocardium, myosin filaments, and collagen fibers [14–16], SHG microscopy can image cardiomyocytes and interstitial collagen fibers quickly and accurately without exogenous labeling. In addition, as an endogenous contrast, tissue autofluorescence (AF) provides a convenient way to identify specific biological structures as a reference.

In this work, we demonstrated that multimodal stimulated emission depletion (STED) microscopy could be used to conduct multi-scale and multi-aspect characterization of the cardiomyocyte microenvironment in the myocardium. A multimodal STED microscope was built, consisting of two-channel SHG, two-color confocal, and two-color STED imaging modalities. BM structures in murine myocardial sections were immunofluorescence (IF) labeled and characterized by SHG- and AF-facilitated multiplexed imaging in 3D without counterstaining. Within ROIs pre-determined by SHG and AF imaging, BM structures surrounding cardiomyocytes and underlying myocardial capillaries were examined by STED imaging for features beyond the diffraction limit. In addition to qualitative observation, quantitative analysis was conducted on the acquired images to extract measurements to characterize myocardial structures.

## 2. Materials and Methods

### 2.1. Multimodal STED Microscope

**Figure 1.**
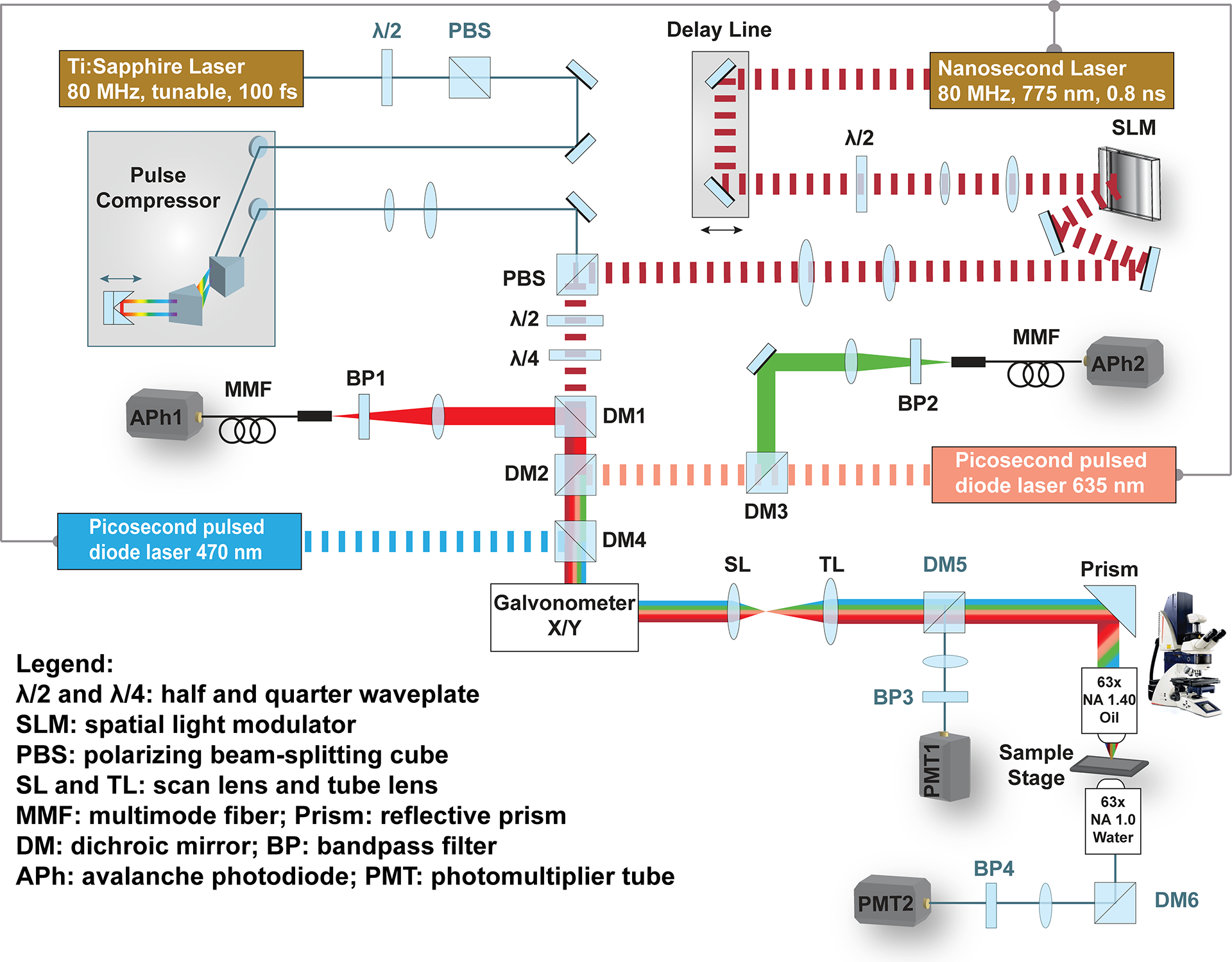
The home-built multimodal STED microscope.

The home-built multimodal STED microscope (Figure 1) was built on an upright commercial microscope (DM6000 CFS, Leica Microsystems, Wetzlar, Germany). This Leica microscope was equipped with brightfield illumination, epi-fluorescence illumination (X-Cite^®^ 120, Excelitas Technologies Corp., Waltham, USA), filter sets (11513825, Leica Microsystems, Wetzlar, Germany; S-001454 and S-405896, Semrock, Rochester, USA), eyepieces, and a monochrome camera (AxioCam HSm, Carl ZEISS Microscopy, Jena, Germany) for routine sample observation. Switching between brightfield, widefield fluorescence, and laser scanning modalities was controlled by Micro-manager [17]. Laser scanning and image acquisition were controlled by SciScan [18] (Scientifica, Clarksburg, USA).

A 775 nm nanosecond pulsed laser (MPB Communications Inc., Montreal, Canada) was holographically modulated by a phase-only spatial light modulator (PLUTO NIR-2, HOLOEYE Photonics AG, Berlin, Germany) to generate a donut-shaped STED focus. A quarter-wave plate and half-wave plate pair were used to obtain a circular polarization of the depletion beam at the entrance pupil of the objective. Two picosecond pulsed lasers (470 nm and 635 nm, LDH Series, PicoQuant, Berlin, Germany) were synchronized to the depletion laser, serving as excitation lights. The depletion and two excitation lights were combined by dichroic mirrors (Semrock DM2: FF662-Di01, DM4: Di02-R488; AVR Optics, Fairport, United States). The scanning unit consisted of an XY two-axis galvanometer set (8310K, Cambridge Technology, Novanta Photonics, Boston, USA), a scan lens (89683, Edmund Optics, Barrington, USA), and a tube lens (ITL200, Thorlabs, Newton, USA). Beams were directed to the entrance pupil of an HCX Plan Apo 63x oil NA 1.40 objective (Leica Microsystems) through the side port of the Leica microscope. For the confocal detection of green fluorescence (CH1), the emission light was spectrally separated from the excitation lights and red fluorescence by dichroic mirrors (DM4, DM2, DM3: FF624-Di01; Semrock), filtered by a bandpass filter (FF01-525/50, Semrock), coupled into a multimode fiber (Thorlabs), and detected by a single photon counting module (SPCM-AQRH-13, Excelitas Technologies Corp., Waltham, United States). For the confocal detection of far-red fluorescence (CH2), the emission light was spectrally separated from the excitation lights and green fluorescence by dichroic mirrors (DM4, DM2, DM1: FF735-Di01; Semrock), filtered by a bandpass filter (690/50, Chroma, Bellows Falls, USA), coupled into a multimode fiber (Thorlabs), and detected by a single photon counting module (SPCM-AQRH-13, Excelitas Technologies Corp.).

A femtosecond mode-locked Titanium: Sapphire laser (Tsunami^®^, Spectra-Physics, Milpitas, USA) with a ~100 fs pulse duration and an 80 MHz repetition rate was integrated into the system as the SHG-excitation light. Before entering the confocal/STED scanning unit, the femtosecond laser was fed into a home-built prism-pair pulse compressor to maintain its short pulse duration by compensating the positive group velocity dispersion introduced by optical elements. A polarizing beamsplitter combined SHG-excitation and STED beams. Backward-propagating SHG (B-SHG) was collected by the imaging objective, spectrally separated by a dichroic mirror (DM5: FF458-Di02, Semrock), filtered by a glass filter (FGB37, Thorlabs) and a bandpass filter (FF01-405/10, Semrock), then detected by a PMT (PMTSS, Thorlabs). Forward-propagating SHG (F-SHG) was collected by a water immersion objective (Plan-Apochromat, 63x, NA 1.0, M27, Carl ZEISS Microscopy, Jena, Germany), deflected by a dichroic mirror (DM6: FF580-FDi01, Semrock), filtered by a glass filter (FGB37, Thorlabs) and a bandpass filter (FF01-405/10, Semrock), then detected by a PMT (H7422-40, Hamamatsu Photonics, Shizuoka, Japan).

### 2.2. Myocardial Sections

All procedures on Sprague–Dawley rats (protocol number AUP2019-048) and C57BL/6 mice (protocol number IACUC-2019-00868) were approved by the Institutional Animal Care and Use Committee (IACUC) of Clemson University and the Medical University of South Carolina. Hearts were harvested from Sprague–Dawley rats or C57BL/6 mice. Ventricles were isolated from the hearts, fixed in freshly prepared 4% (*w*/*v*) paraformaldehyde, cryoprotected by sucrose solution, then snap-frozen in liquid nitrogen-cooled isopentane. Frozen blocks were sectioned and mounted on microscope slides (VWR International, Radnor, USA).

### 2.3. Immunofluorescence (IF)

Heat-induced antigen retrieval was performed by incubating sections in sodium citrate pH 6.0 at 95 °C for 20 min. Sections were blocked in 2% (*w*/*v*) bovine serum albumin with 0.3% Triton X-100 at room temperature, then incubated with anti-laminin (ab11575, Abcam, Cambridge, UK), β1 integrin (ab179471, Abcam), collagen type III (600-401-105, Rockland, Philadelphia, USA), or collagen type IV (ab179471, Abcam) at 4 °C overnight. Sections were incubated with STAR RED (Abberior, Gottingen, Germany) at room temperature for 3 h. After staining, sections were mounted in custom-made Mowiol medium and coverslipped. Primary and secondary antibodies were diluted in the blocking buffer. Negative primary antibody controls were performed to confirm that the staining signal was from the detection of target antigens. The antibodies used in this study are summarized in Table S1.

### 2.4. Imaging Procedures

For SHG imaging, the wavelength of the SHG-excitation laser was tuned to 810 nm to excite filamentous structures in myocardial sections, i.e., myosin filaments and collagen fibrils/fibers. SHG-excitation laser power was set at ~20 mW, measured at the entrance pupil of the objective.

The excitation laser at 470 nm was used for AF imaging to excite endogenous fluorophores and fixative-induced fluorescence. AF images were acquired at CH1 with an emission range of 525 ± 25 nm.

The excitation laser at 635 nm was used for confocal imaging of STAR-RED-labeled protein. IF images were acquired at CH2 with an emission range of 690 ± 25 nm. STED imaging of STAR-RED-labeled protein was conducted on ROIs pre-determined by SHG or AF imaging. The depletion laser power was set at ~170 mW at the entrance pupil of the objective.

### 2.5. Image Processing and Reconstruction

Raw images (1024 × 1024 or 512 × 512 pixels, 16-bit grayscale) underwent frame averaging, then Gaussian or Wiener filtering. No further processing was applied.

Z-stacks were aligned to correct sample drift during Z-stack imaging using a Fiji plugin, Linear Stack Alignment with SIFT [19]. Translational sample drift was corrected using the default settings of point detector, feature descriptor, and consensus filter. Aligned stack images were post-processed by a custom MATLAB script to generate masked images. Image binarization (global or adaptive) was optimized using the Image Segmenter App in MATLAB. The aligned and masked stack images were used for 3D reconstruction in Imaris 9.0.1 (Oxford Instruments, Abingdon, UK).

### 2.6. Sarcomere Length Measurements

**Figure 2.**
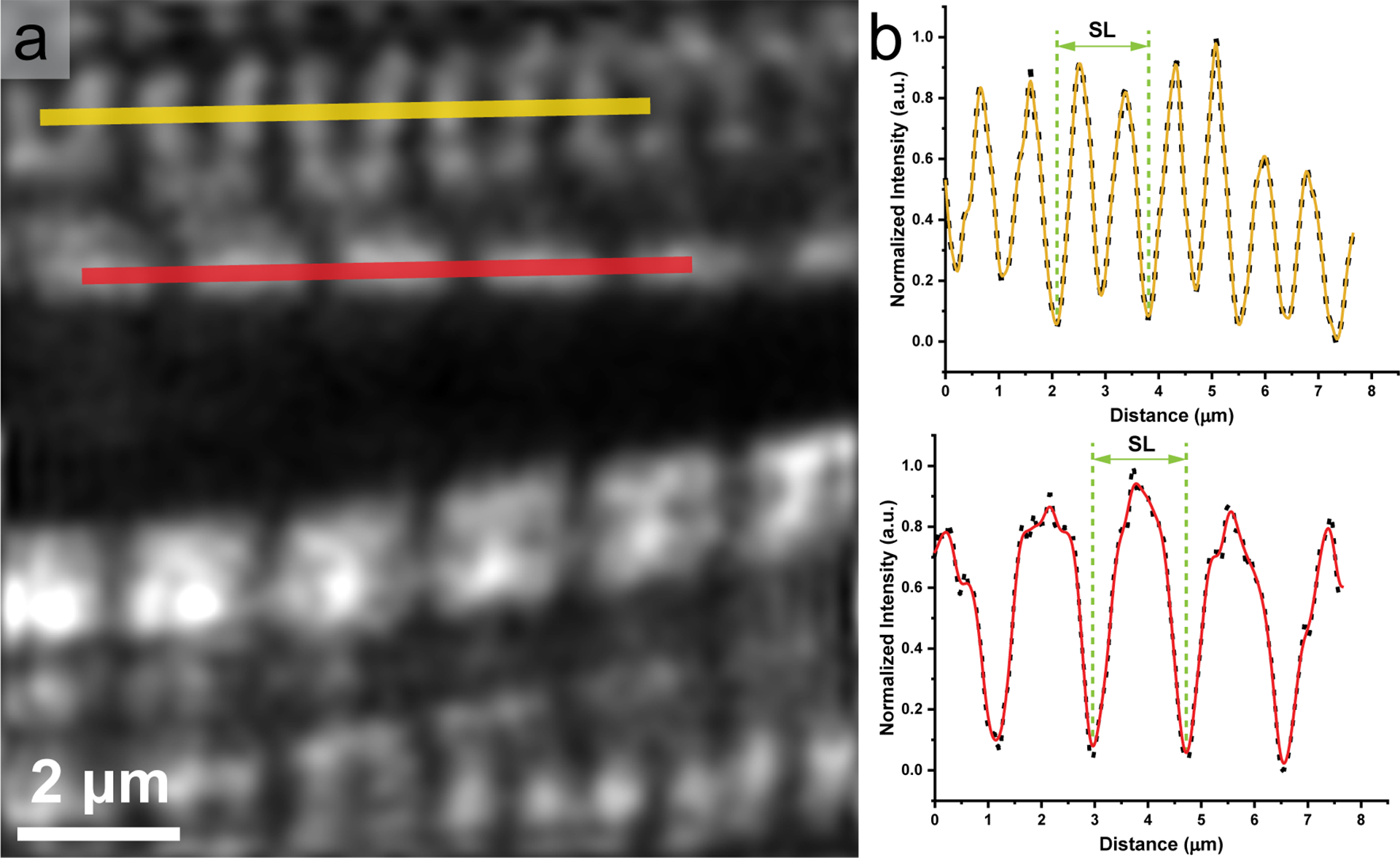
(**a**) Double-band and single-band myosin lattice in a myocardial section of adult C57BL/6 mouse. Scale bar, 2 μm. (**b**) Sarcomere length (SL) is marked along double-band (yellow) and single-band (red) myosin lattice in line profiles.

Sarcomere length was measured from F-SHG images as myosin filaments produced pronounced F-SHG contrast. Two patterns of myosin lattice were commonly observed in murine myocardial sections: double-band and single-band. Only the rod domain of myosin filaments can emit an SHG signal, while M-lines within A-bands have no SHG contrast [20]. When two sides of myosin filaments are resolved, the myosin lattice features a double-band pattern; if not, the myosin lattice shows a single-band pattern (Figure 2a). By plotting line profiles along the direction of myofibrils, sarcomere length was obtained by measuring the distance between two adjacent intensity local minima, excluding local minima within A-bands (Figure 2b).

### 2.7. Capillarization-Related Measurements

Segmentation of cardiomyocytes and capillaries was carried out using a Segment-Anything-Model-based instance segmentation tool, AutoQC [21]. The following capillarization-related measurements were calculated from segmented masks for each ROI:

- Capillary density normalized to FOV (μm^−2^) was defined as capillaries per FOV area and calculated as the total number of masks with a capillary category divided by the FOV.
- Capillary density normalized to cardiomyocyte area (μm^−2^) was defined as capillaries per cardiomyocyte area and calculated as the total number of masks with a capillary category divided by the total area of masks with a cardiomyocyte category.
- The capillary-to-cardiomyocyte ratio was calculated by dividing the number of masks in a capillary category by the number of masks in a cardiomyocyte category.

## 3. Results

### 3.1. Multiplexed Imaging of Myocardial Structures in 3D

**Figure 3.**
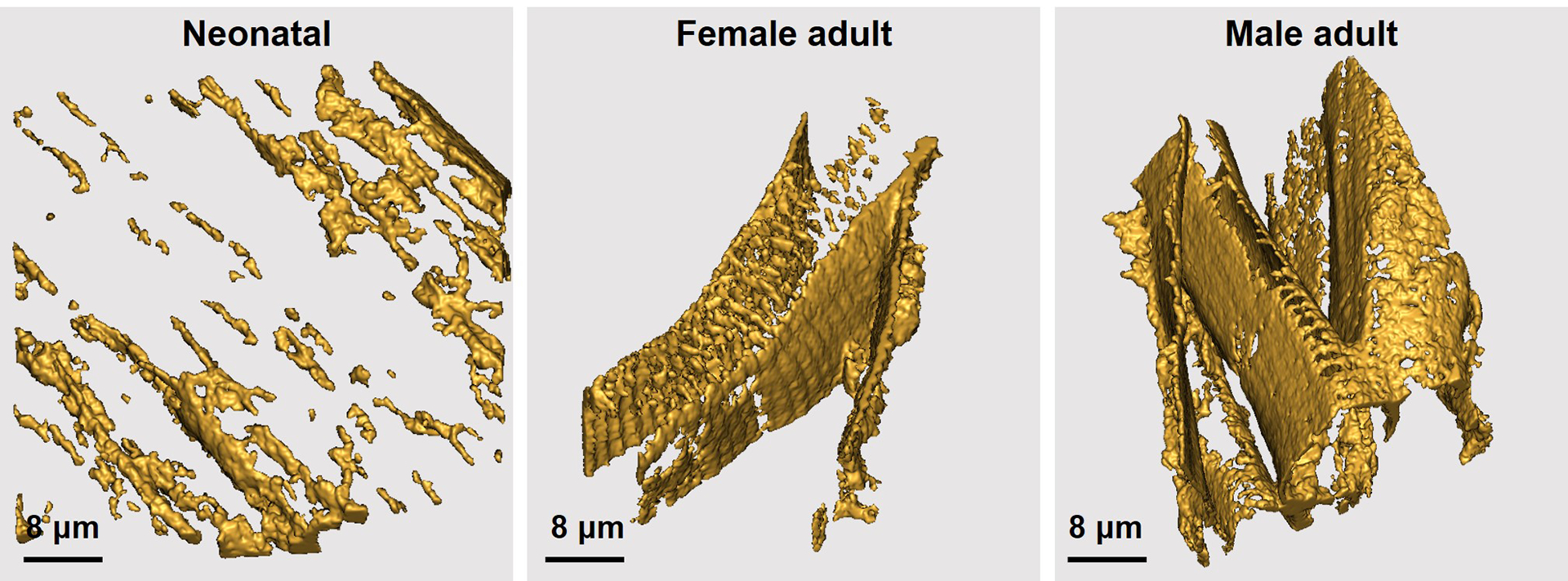
Reconstructed models show laminin-denoted BM surrounding cardiomyocytes in neonatal, female adult, and male adult rat myocardium. Z-step, 0.25 μm. Stack depth, 10.5 μm (neonatal); 11 μm (female adult); 23.25 μm (male adult). Scale bars, 8 μm.

To illustrate the necessity of multiplexed imaging, single-color confocal imaging was performed on rat myocardial sections. Laminin, a major BM component, was IF stained to visualize BM structures. Immature BM structures are evident in the reconstructed model of day-3 neonatal myocardium, with an incomplete enclosure appearance (Figure 3, neonatal). In contrast, BM structures in female and male adult rat myocardium show a dense and continuous layer enclosing individual cardiomyocytes (Figure 3, female and male adult). Furthermore, laminin shows noticeable interactions with the cardiomyocyte sarcolemma, as evidenced by wave-like protrusions towards the cardiomyocyte on the BM surface (Figure 3, female adult). This observation aligns with the well-accepted understanding that laminin interacts with cell surface receptors, such as integrins and dystroglycan [22–24]. Only laminin-denoted BM structures are visible in single-color IF images, limiting further interpretation and analysis. As sections acquired from multiple animal sources were used in this study, Table S2 summarizes sample sources for each image and related result presented in the result section.

**Figure 4.**
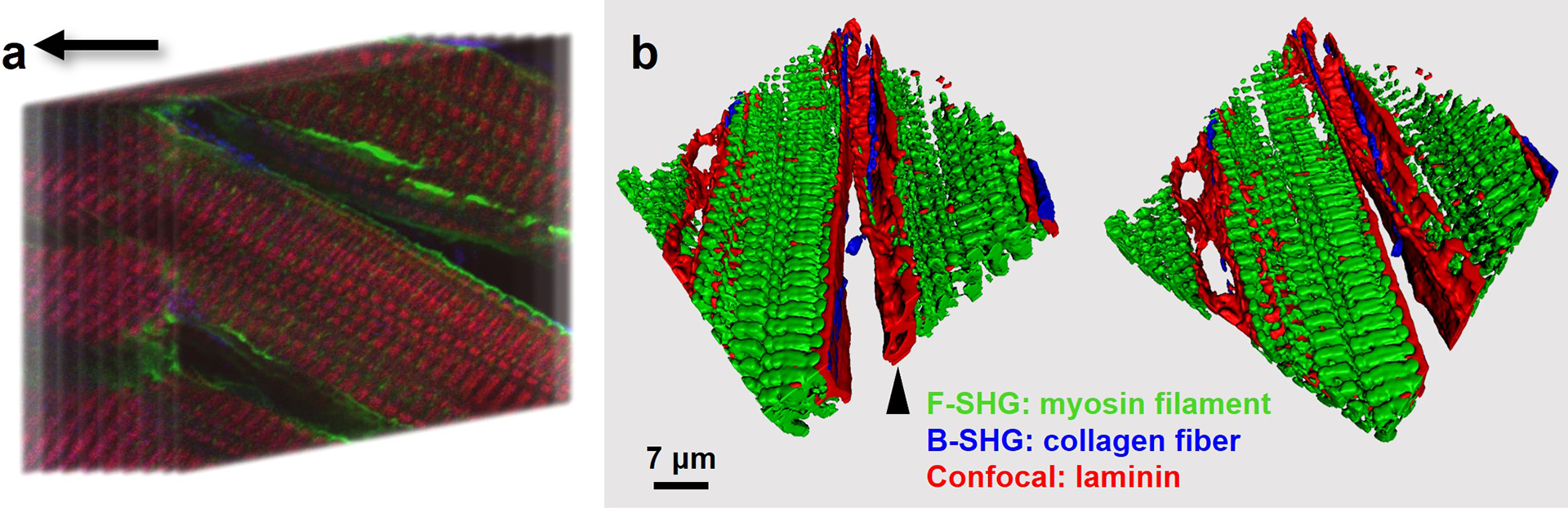
(**a**) Schematic of Z-stack confocal fluorescence and SHG imaging. (**b**) 3D reconstructed model of a female adult rat myocardial section shows myosin lattice (green) in cardiomyocytes, laminin-denoted BM (red), and interstitial collagen fibers (blue). A capillary adjacent to the cardiomyocyte is marked by an arrowhead. Z-step, 0.25 μm. Stack depth, 10 μm.

By integrating with SHG microscopy, complementary F- and B-SHG channels can capture myosin filaments and fibrillar collagen, providing reference structures without the need for exogenous labeling. Myosin filaments aid in the localization of cardiomyocytes, while fibrillar collagen helps interpret the interstitial matrix in the extracellular domain. Figure 4 shows a reconstructed 3D model with cardiomyocytes (denoted by myosin lattice in F-SHG), BM (denoted by laminin in confocal), and interstitial matrix (denoted by thick collagen fibers in B-SHG). This multiplexed model reveals wave-like protrusions on the BM surface extending towards I-bands around Z-lines, forming contacts with the cardiomyocyte sarcolemma. In the myocardium, BM also underlies endothelial cells lining microvessels. A capillary adjacent to the cardiomyocyte is enclosed by a laminin sheet, as evidenced by the absence of an SHG signal inside the lumen (Figure 4b, arrowhead). Aided by SHG contrast, laminin distribution was better defined. Without counterstaining, the structural interactions between BM and the cardiomyocyte sarcolemma were interpreted in 3D with identifiable cardiomyocytes, pericellular BM, and the interstitial matrix.

**Figure 5.**
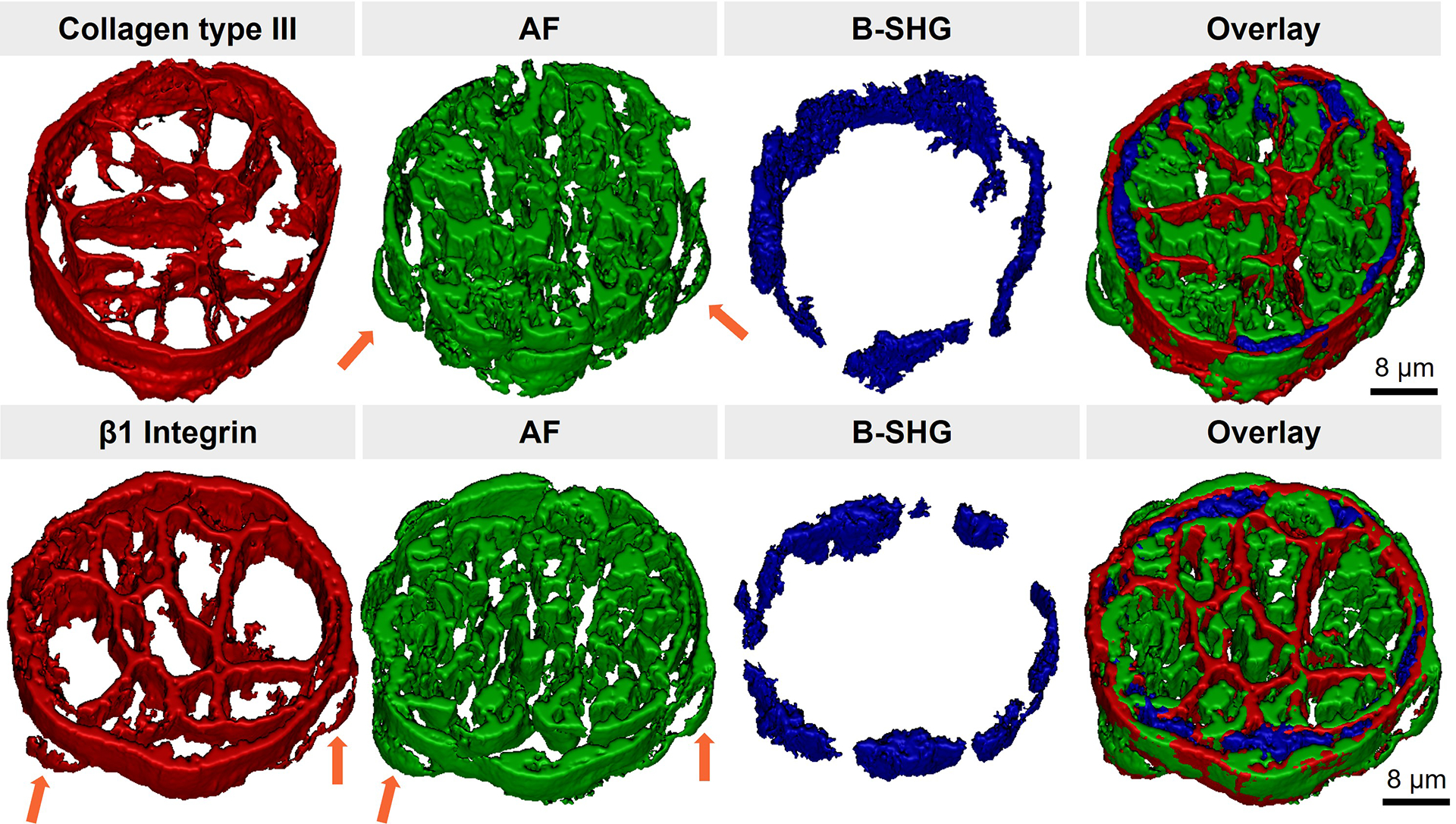
Reconstructed models show the endocardium (in AF), cardiomyocytes (in AF), and interstitial matrix (in B-SHG) within papillary muscle cross-sectioned areas. Collagen type III surrounds individual cardiomyocytes and the cardiomyocyte bundle (top row). β1 integrin distributes on cardiomyocytes and endothelial cells (bottom row). Arrowheads highlight nuclei of squamous endothelial cells within the endocardium. Z-step, 0.5 μm. Stack depth, 13 μm (top row); 11.5 μm (bottom row).

SHG imaging detects intrinsic SHG sources in the myocardium in a label-free manner, providing an elegant approach for the background-free localization of myocardial structures. Tissue AF, arising from endogenous fluorophores and fixative-induced fluorescence, can also be used to facilitate the interpretation of myocardial structures in tissue samples [25]. AF imaging can be conducted utilizing the same setup as confocal IF imaging. Thus, AF imaging not only inherits an optical-sectioning capability but also intrinsically complements IF imaging. We have identified autofluorescent structures in cardiomyocytes as mitochondria, while the endothelium of microvessels and the endocardium also produce observable AF contrast [25]. To demonstrate 3D multiplexed imaging utilizing the contrast mechanisms of IF, AF, and SHG, β1 integrin (transmembrane protein) or collagen type III (ECM structural protein) in mouse myocardial sections were IF stained. The reconstructed models in Figure 5 show cardiomyocytes surrounded by the endocardium in cross-sectioned papillary muscle samples. Papillary muscle is the protrusion of cardiac muscle into ventricular chambers, where it attaches to atrioventricular valves to prevent regurgitation of ventricular blood. Mitochondria clusters in cardiomyocytes and the endothelium of the endocardium were captured by AF imaging, while interstitial collagen fibers were captured by SHG imaging. Collagen type III, a major structural protein, along with collagen type I form a dense interstitial matrix in the myocardium. Collagen type III encloses individual cardiomyocytes, forming the endomysium, and further surrounds cardiomyocyte bundles, forming the perimysium [26]. Compared to the IF of collagen type III, SHG imaging only detected thick collagen fibers in the perimysium surrounding cardiomyocyte bundles (Figure 5, top row). Due to the diffraction-limited resolution, the perimysial layer cannot be separated from the endocardium. Fortunately, some clues can be found using endothelial cell nuclei as a reference, as nuclei have no AF contrast [25] (see also Figure S1). The absence of collagen type III on the surface of two endothelial cells (Figure 5, arrows in top row) provides direct evidence that the endocardium forms an outer layer enclosing the perimysium of the papillary muscle. In contrast, β1 integrin, a conserved subunit of integrins expressed on both cardiomyocytes and endothelial cells, demonstrates a distribution on the surface of endothelial cells within the endocardium (Figure 5, arrows in bottom row).

### 3.2. Super-Resolution Imaging of Basement Membrane in the Myocardium

As previously demonstrated, diffraction-limited microscopy techniques, such as conventional confocal fluorescence and SHG microscopy, are unable to resolve fine details of myocardial structures, impeding accurate characterization of target structures and evaluation of protein distribution. Within the myocardium, cardiomyocytes and capillaries are densely packed, highly ordered, and enclosed by the endomysium. The BM, a thin sheet-like structure, surrounds cardiomyocytes and underlies endothelial cells in the pericellular domain. STED imaging of collagen type IV, a major BM protein, was conducted to characterize features of BM structures in myocardial sections beyond the diffraction limit.

**Figure 6.**
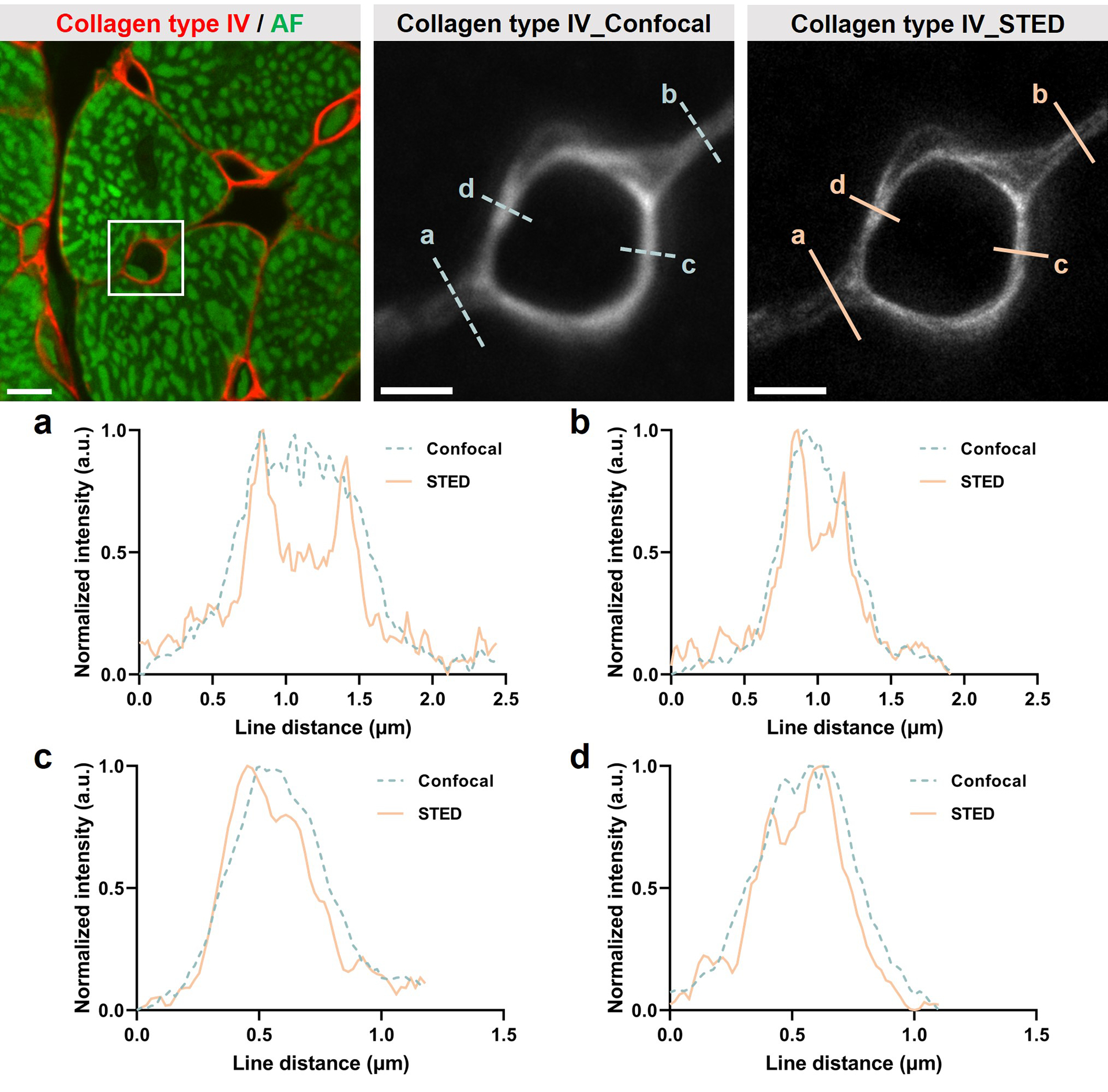
AF imaging enabled searching potential ROIs and differentiating cardiomyocytes from capillaries in a fast and label-free manner. A representative ROI (white box in the zoom-out image) was identified, and then STED imaging was performed within a zoom-in FOV. Line profiles (**a**) and (**b**) show that BM structures of adjacent cardiomyocytes can be resolved by STED. Line profiles (**c**) and (**d**) show that the BM structures of a capillary and its neighboring cardiomyocytes were partially resolved by STED. Scale bars, 5 μm (zoom out) and 2 μm (zoom in).

AF imaging was utilized to search tissue samples, identify potential ROIs, and differentiate cardiomyocytes from capillaries. Using AF contrast, mitochondria clusters indicate the presence of cardiomyocytes, while the endothelium and blood cells within the lumen show intramyocardial capillaries. Once an ROI containing both cardiomyocyte BM and capillary BM structures was identified, optical zoom-in was carried out via SciScan. STED imaging was performed on the zoomed ROI for super-resolution characterization. Using confocal and STED imaging we observed a representative ROI containing the borders of two adjacent cardiomyocytes and a capillary in between them (Figure 6). Confocal imaging failed to resolve the BM structures on the border of two adjacent cardiomyocytes. In contrast, STED imaging not only resolved the BM structures of adjacent cardiomyocytes but also revealed the BM structure of their neighboring capillary.

### 3.3. Quantitative Assessment of Myocardial Structures in Multimodal Microscopy

We have demonstrated that the home-built multimodal STED microscope can achieve multi-aspect and multi-scale visualization of myocardial structures at the tissue level. In addition to qualitative observation, image analysis of acquired images allows for quantitative assessment of myocardial structures. We analyzed cardiomyocyte sarcomeres and myocardial capillary networks as examples.

Sarcomeres are contractile units in cardiomyocytes that generate active and passive force. The distance between adjacent Z-lines is defined as sarcomere length. Changes in sarcomere length are associated with force generation by the Frank–Starling relationship (length-dependent contractility). Characterizing sarcomere length provides an effective approach to monitoring structural and functional alterations of cardiomyocytes [27]. Sarcomere length was measured on SHG images acquired by the home-built microscope. Myocardial sections were collected from three 5-month C57BL/6 female littermates. For each mouse, five randomly selected sections were imaged. A total of 34 measurements were obtained by analyzing SHG images using a custom-written MATLAB script. Sarcomere length in the left ventricular myocardium of adult female mice demonstrated a value of 1.84 ± 0.078 μm (*n* = 34). This result is consistent with the sarcomere length measured by TEM image analysis of wild-type mice with a mixed 129Sv/C57BL/6 genetic background (1686.0 ± 123.4 nm) [28].

**Figure 7.**
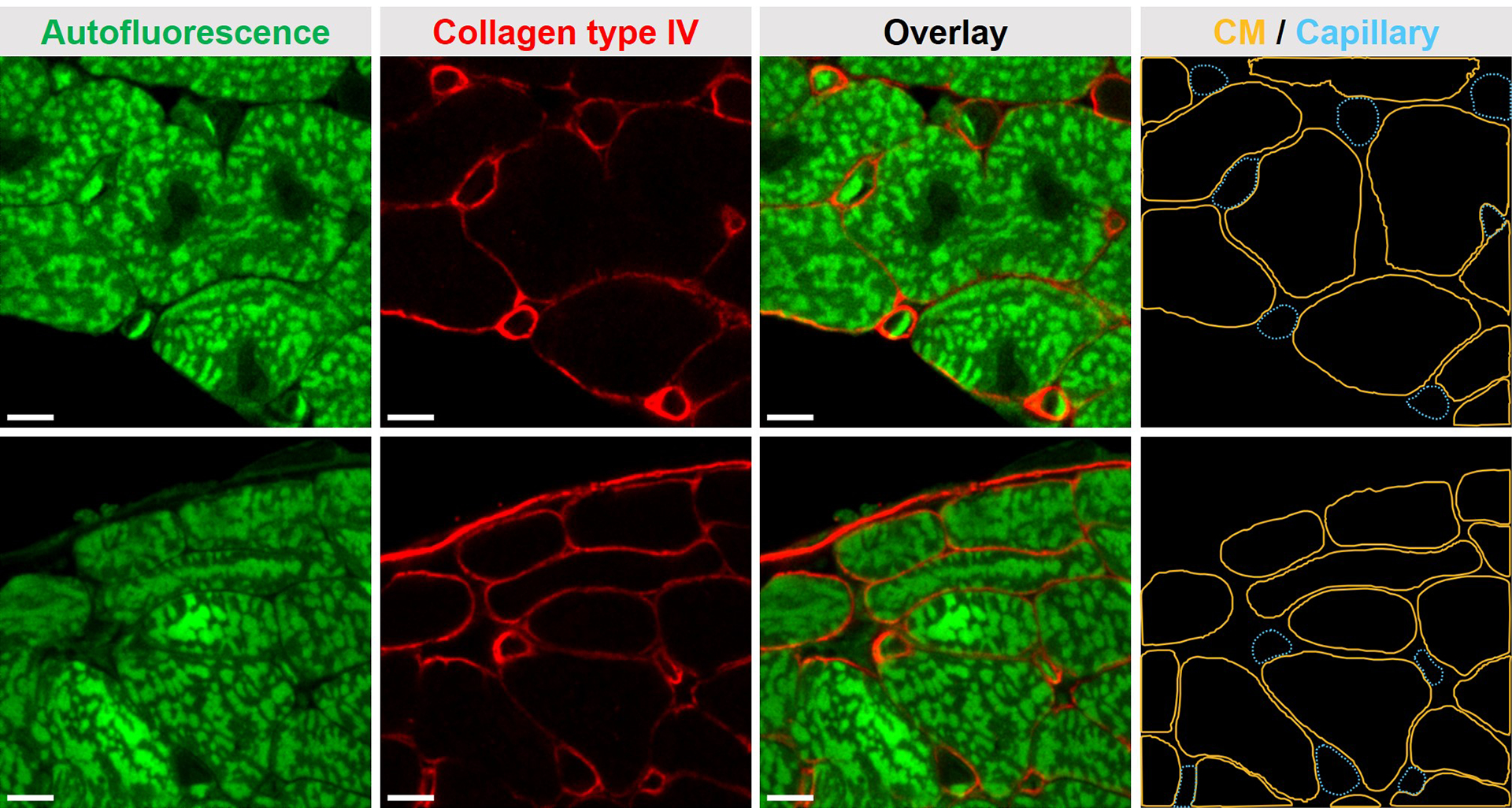
AF-facilitated IF imaging enabled the quantitative assessment of the capillary network in myocardial sections. Segmented masks of cardiomyocytes (CMs, solid orange line) and capillaries (dashed blue line) were used to calculate capillarization-related measurements. Scale bars, 5 μm.

Capillaries play an important role in maintaining cardiac function by regulating local blood perfusion and metabolic exchange. Vascular endothelial dysfunction and cardiac capillary rarefaction have been reported to be correlated with heart disorders [29–31]. Capillary density is an effective measurement of microcirculation, reflecting microvascular blood perfusion and oxygen consumption in tissues. Measurements of myocardial capillary density require microscopic examination of myocardial sections. AF-facilitated IF imaging of collagen type IV was conducted for the quantitative assessment of the myocardial capillary network (Figure 7). Tissue AF imaging was utilized to differentiate cardiomyocytes from capillaries in myocardial sections. Three randomly selected myocardial sections were collected from a 5-month C57BL/6 female mouse. A total of 10 ROIs at cardiomyocyte cross-section areas were imaged. Quantitative analysis demonstrated a capillary density of 0.00463 ± 0.00117 μm^−2^ when normalized to FOV, and 0.00574 ± 0.00141 μm^−2^ when normalized to the total area occupied by cardiomyocytes. The capillary-to-cardiomyocyte ratio was 0.834 ± 0.220.

## 4. Discussion and Conclusions

To properly facilitate the examination of cell structures and tissue morphology, observable AF contrast in tissue samples should be identified. We have identified major autofluorescent structures in formaldehyde-fixed myocardial sections by IF staining, with an emphasis on typical histological structures within the myocardium. Aldehyde fixatives should introduce similar AF contrast in myocardial sections, as they share the same fixation mechanism. Amine condensation occurs when aldehyde groups react with proteins, yielding fluorescent products [32]. AF contrast should be validated in myocardial samples prepared using a different protocol to ensure accurate interpretation when employing AF-facilitated imaging.

Different microscopies utilize different contrast mechanisms and require specific sample preparation procedures. Both confocal and STED imaging rely on IF staining of target proteins with fluorescent dyes. Unlike AF/IF contrast, SHG contrast prefers minimal sample processing to preserve native structures, thus avoiding the degradation of intrinsic SHG sources. When performing multicolor staining or using fluorescent dyes with excitation/emission spectra similar to tissue AF, strong AF may interfere with the IF of target proteins. This issue can be more problematic under low SNR conditions, such as proteins labeled by fluorescent dyes with a low quantum yield and samples exposed to intense illumination during STED imaging. As such, it is essential to investigate and optimize sample preparation to achieve a balanced image quality when performing multi-aspect characterization using multimodal microscopy.

STED microscopy inherits the advantages of confocal microscopy, providing a cost-effective approach for 3D characterization compared to EM techniques. Among super-resolution microscopy techniques, STED microscopy is more suitable for tissue imaging. STED imaging does not rely on computational estimation and reconstruction to achieve diffraction-unlimited resolution. Its subdiffractional resolution is associated with subdiffractional excitation. The achievable spatial resolution depends on the depletion power. Accordingly, BM structures on the border of cardiomyocytes and neighboring capillaries can be fully resolved by increasing STED laser power. It should be noted that the resolution improvement comes at the cost of a low photon budget.

The two-color STED capability of the home-built microscope holds the potential as a powerful tool for characterizing the interactions between cardiomyocytes and their microenvironment. Evidence from patients and animal models has demonstrated that abnormal interactions between cardiomyocytes and their surroundings are pathogenic and associated with cardiomyopathy [33–35]. By two-color IF staining of cell membrane receptors (integrins, dystroglycan, or discoidin domain receptors) and ECM proteins, two-color STED can be used to observe physical binding behaviors between receptors on cardiomyocyte sarcolemma and ECM ligands. By conducting a colocalization analysis of two-color STED images, the co-distribution of two proteins can be evaluated as a direct measurement of interactions. This quantitative assessment will determine whether the two proteins are associated with the same site.

We have demonstrated that multimodal STED microscopy can be used for the multi-scale and multi-aspect characterization of cardiomyocyte microenvironments at the tissue level. Various myocardial structures, such as cardiomyocytes, myocardial capillaries, and interstitial matrix, can be identified using SHG and AF contrast, eliminating the need for counterstaining and simplifying sample preparation. Moreover, myocardial structures, such as sarcomeres (contractile units in cardiomyocytes) and the intramyocardial capillary network (the local circulation system in the myocardium), can be quantitatively assessed by analyzing the acquired images.

## Supplementary Material

Supplementary document

## Data Availability

All datasets and MATLAB scripts are available from the corresponding authors upon reasonable request.
