# Supplementary material for: In Situ Structural Characterization of Cardiomyocyte Microenvironment by Multimodal STED Microscopy
Source: Photonics. Author manuscript; Available in PMC 2024 Oct 10. (PMC11466047; doi:10.3390/photonics11060533)
Supplement: Supplementary document [file NIHMS2026361-supplement-Supplementary_document.docx]

**Supplementary Table 1.**

List of antibodies used in the study.

| **Primary antibody** | | | |
| --- | --- | --- | --- |
| **Target** | **Product number** | **Vendor** | **Dilution** |
| Laminin | ab11575 | Abcam | 1:200 |
| β1 integrin | ab179471 | Abcam | 1:200 |
| Collagen type III | 600-401-105 | Rockland | 1:200 |
| Collagen type IV | ab179471 | Abcam | 1:200 |
| **Secondary antibody** | | | |
| **Target** | **Product number** | **Vendor** | **Dilution** |
| Rabbit IgG | STRED-1002 | Abberior | 1:500 |

**Supplementary Table 2.**

List of animals where ventricular sections were collected and used in this study.

| **Immunostaining of laminin (Figure 3)** |
| --- |
| Day-3 neonatal Sprague-Dawley rat, a 10 μm-thick section |
| 4-week pregnant female Sprague-Dawley rat, a 20 μm-thick section |
| 4-week male Sprague-Dawley rat, a 20 μm-thick section |
| **Immunostaining of laminin (Figure 4)** |
| 4-week pregnant female Sprague-Dawley rat, a 20 μm-thick section |
| **Immunostaining of β1 integrin or collagen type III (Figure 5)** |
| 5-month C57BL/6 female mouse, 8 μm-thick sections |
| **Immunostaining of collagen type IV (Figure 6)** |
| 5-month C57BL/6 female mouse, an 8 μm-thick section |
| **Quantification of sarcomere length** |
| 3 littermates of 5-month C57BL/6 female mice  5 randomly selected 8 μm-thick sections from each mouse |
| **Quantification of capillary density** |
| 3 randomly selected 8 μm-thick sections from a 5-month C57BL/6 female mouse |

**Supplementary Figure 1.**


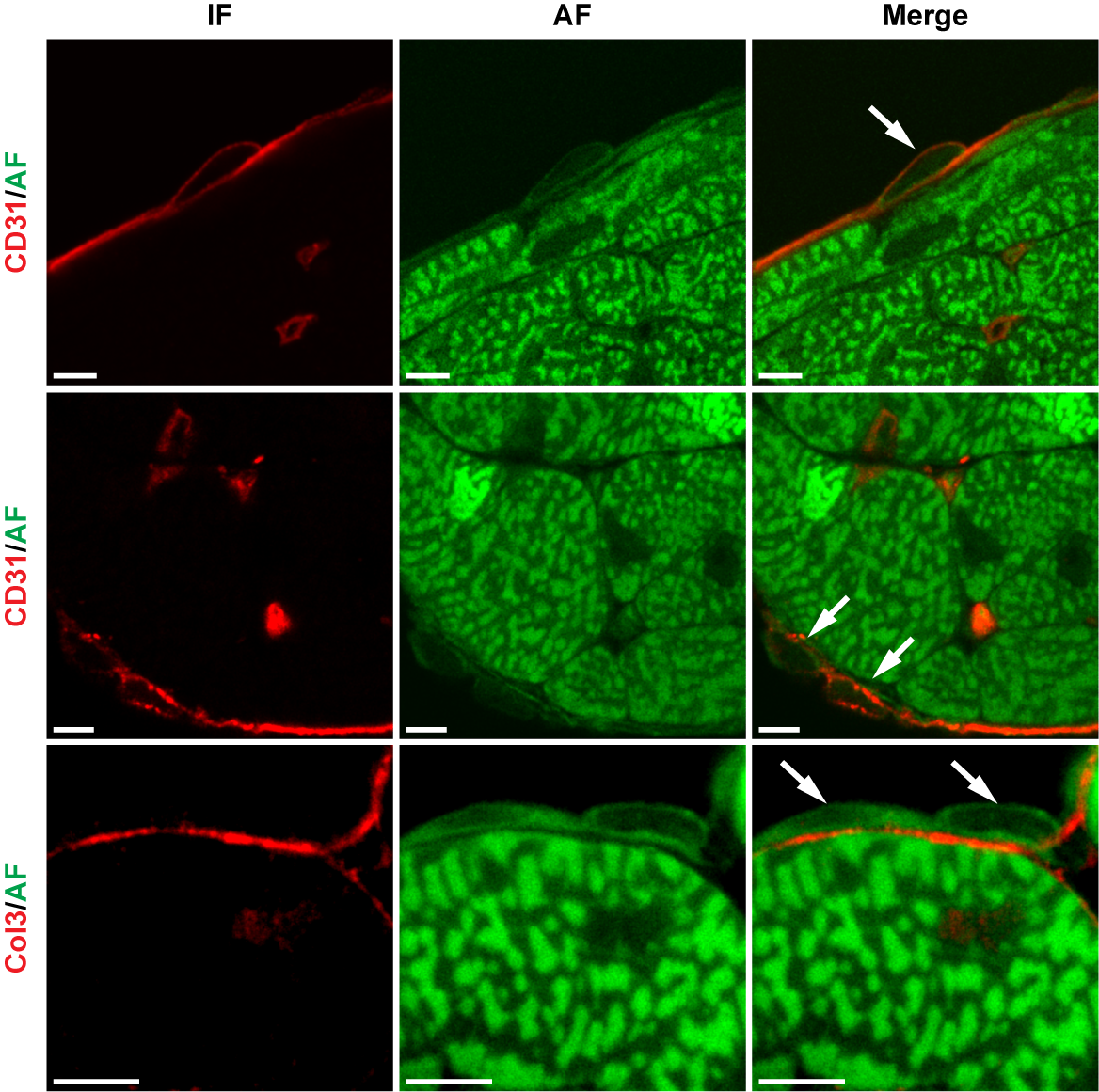


Representative confocal AF and IF images show that the nuclei of simple squamous endothelial cells within the endocardium lack AF contrast. Immunostaining of CD31 (1:100 dilution, DIA-310, Dianova) detects endothelial cells lining up intramyocardial capillaries and the innermost layer of heart chambers. Immunostaining of collagen type III captures the interstitial collagen network surrounding individual cardiomyocytes and cardiomyocyte bundles. Arrows highlight the nuclei of squamous endothelial cells within the endocardium. Scale bars, 5 μm.
